# The association between chronic conditions, COVID-19 infection, and food insecurity among the older US adults: findings from the 2020–2021 National Health Interview Survey

**DOI:** 10.1186/s12889-023-15061-8

**Published:** 2023-01-27

**Authors:** Jiahui Cai, Aurelian Bidulescu

**Affiliations:** Department of Epidemiology and Biostatistics, Indiana University Bloomington, Bloomington, IN USA

**Keywords:** Household food insecurity, Chronic conditions, COVID-19 pandemic, Older adults, National health interview survey

## Abstract

**Background:**

This study aims to examine how the presence of chronic conditions or positive COVID-19 infection (as exposures) is related to food insecurity (as an outcome) in the older population and whether there is a dose–response relationship between the number of chronic conditions and the severity of food insecurity.

**Methods:**

Cross-sectional data of 17,977 older adults (≥ 65 years) from the 2020–2021 National Health Interview Survey were analyzed. Chronic conditions included physical health conditions (i.e., arthritis, coronary heart diseases, hypertension, stroke, prediabetes, diabetes, asthma, chronic obstructive pulmonary disease, and disability) and mental health conditions (i.e., anxiety and depression disorder). COVID-19 infection status was determined by a self-reported diagnosis of COVID-19. Household food insecurity was measured using the 10-item US Department of Agriculture (USDA) Food Security Survey Module with a 30-day look-back window. Multinomial logistic regression models were used to examine the association between health conditions and food insecurity controlling for socio-demographic factors.

**Results:**

Our results indicated that 4.0% of the older adults lived in food-insecure households. The presence of chronic conditions was significantly associated with higher odds of being food insecure independent of socio-demographic factors (AOR ranged from 1.17 to 3.58, all *p* < 0.0001). Compared with participants with 0–1 chronic condition, the odds of being (low or very low) food insecure was 1.09 to 4.07 times higher for those with 2, or ≥ 3 chronic conditions (all *p* < 0.0001). The severity of food insecurity significantly increased as the number of chronic conditions increased (*p* for trend < 0.0001). Besides, COVID-infected participants were 82% more likely to be very low food secure than the non-infected participants (AOR = 1.82, 95% CI: 1.80, 1.84).

**Conclusions:**

The presence of chronic conditions or positive COVID-infection is independently associated with household food insecurity. Clinical health professionals may help identify and assist individuals at risk of food insecurity. Management and improvement of health conditions may help reduce the prevalence and severity of food insecurity in the older population.

**Supplementary Information:**

The online version contains supplementary material available at 10.1186/s12889-023-15061-8.

## Introduction

Food insecurity is characterized by limited or uncertain access to adequate food [1]. Based on the US Department of Agriculture (USDA) report, 13.8 million households (10.5%) of all US households were food insecure in 2020 [2]. Statistics from Feeding America indicated that 5.2 million (6.8%) adults aged 60 years and older in the U.S. lived in food-insecure households in 2020 and 2.0 million (2.6%) older adults lived in households with very low food security [3]. Compared to 2001, the prevalence of food insecurity and very low food security for older US adults (≥ 60 years) in 2020 has increased by 29% and 84%, respectively [3]. The prevalence of food insecurity for older people (≥ 65 years) living alone has also increased from 8.3% in 2020 to 9.5% in 2021 [4]. Given that the proportion of the older population is expected to be 21.6% by 2040 in the U.S., the food insecurity issue among this community is concerning [5].

Although previous studies have suggested socioeconomic factors such as education level, household income, household sizes (such as the number of adults or children in the household), and employment status were significantly associated with food insecurity [6, 7], there is limited evidence on how health conditions may affect food insecurity. The presence of chronic conditions may likely increase the vulnerability of food insecurity by limiting individuals’ work ability and employment status and adding household financial burdens due to additional healthcare expenditures. Compared to healthy populations, individuals with chronic conditions (such as diabetes, musculoskeletal conditions, respiratory, digestive, and psychological conditions) were 32% more likely to be unemployed and 11% more likely to retire early [8]. Reduced income due to unemployment or early retirement could further increase the risks of food insecurity [9, 10]. Poorer health conditions may also take up finances that would otherwise be used on food. The presence of chronic medical conditions increased the total healthcare expenditure by 35% to 40% [11] and the costs increased as the number of treated chronic conditions increased [12]. Compared with individuals without hypertension, those with hypertension had $1,920 higher annual healthcare expenditures, 2.5 times higher inpatient costs, double outpatient costs, and over 3 times higher prescription medication expenditures [13]. Considering the financial burden of chronic conditions and the fact that more than 80% of older US adults (≥ 65 years) live with multiple chronic conditions [14], it is necessary to identify whether the presence of those chronic conditions in the older community may render their households more vulnerable to food insecurity.

The coronavirus disease 2019 (COVID-19) pandemic that began in 2020 in the U.S. has significantly affected food systems and contributed to increased food insecurity in the early days of the pandemic [15]. The National Food Access and COVID Research Team (NFACT) reported a 34.7% increase in food insecurity in a state-representative sample at the beginning of the pandemic [16]. Pandemic-induced unemployment and strict quarantine or stay-at-home order increased difficulty in accessing food resources for both the general population and the COVID-19-infected population, while a 3% higher prevalence of food insecurity was observed in the COVID-infected population than the non-infected population after adjusting for age, sex, and employment [17]. One possible explanation is that COVID-19 infection and post-COVID-19 conditions (such as fatigue, chest tightness, myalgia, and mental disorder) may add additional financial burdens related to healthcare expenditures, thus increasing the vulnerability of household food insecurity [18]. Total health care cost was $3,706 (for the commercial cohort) to $10,595 (for the Medicare cohort) significantly higher for COVID-19 patients than for the non-infected populations [19]. However, it is still unclear whether COVID-19 infection is independently associated with an increased likelihood of food insecurity after controlling for socioeconomic factors such as education level, household sizes, and poverty, especially for older adults who are one of the most vulnerable groups to COVID-19 infection [20].

Using the nationally representative data from the 2020 and 2021 National Health Interview Survey (NHIS), we conducted a cross-sectional study to fill in those research gaps. In this study, we aimed to examine 1) the association between individual chronic conditions (including physical and mental conditions) and food insecurity in older adults (≥ 65 years); 2) the association between the number of chronic conditions and food insecurity, and whether there is a dose–response relationship between the number of chronic conditions and severity of food insecurity; 3) the association between COVID-19 infection and food insecurity. Specifically, we considered physical health conditions including arthritis, coronary heart disease (CHD), hypertension, stroke, prediabetes, diabetes, asthma, chronic obstructive pulmonary disease, disability, and mental health conditions including anxiety and depression disorder. Identifying how the presence of those chronic conditions or COVID-19 infection is related to food insecurity may add new insights into factors associated with food insecurity beyond the traditionally well-established socioeconomic characteristics.

## Methods

### Study sample and participants

Data used in this study were derived from 2020 and 2021 NHIS, an ongoing cross-sectional and nationally representative survey which aims to monitor the trends in health, illness, and disability in the U.S. civilian noninstitutionalized population [21]. With stratified clustering sampling, NHIS randomly selected roughly 30,000 households from the randomly selected clusters within 1,689 originally defined geographic areas. Sample adults were then selected from each household [21]. For each cycle, NHIS continuously collected data from January to December throughout the year on both the household level (such as household demographics and food insecurity) and the individual level (such as health conditions and behaviors, health care access and utilization, functioning and disability, and COVID-related data). Due to the impact of the pandemic, the 2020 and 2021 NHIS predominantly collected data using telephone interviews rather than regular in-person visits, which accounted for relatively low response rates during this time (response rates: 50.7% for sampled households and 48.9% for sampled adults in 2020 NHIS and 52.8% for sampled households and 50.9% for sampled adults in 2021 NHIS) when compared to the rates in 2019 before the COVID-19 pandemic (response rates: 61.1% for sampled households and 59.4% for sampled adults in 2019 NHIS) [21]. Quality controls were conducted through monthly checks on response rates, item response times or non-response, telephone usage rates, and other data quality indicators.

**Fig. 1:**
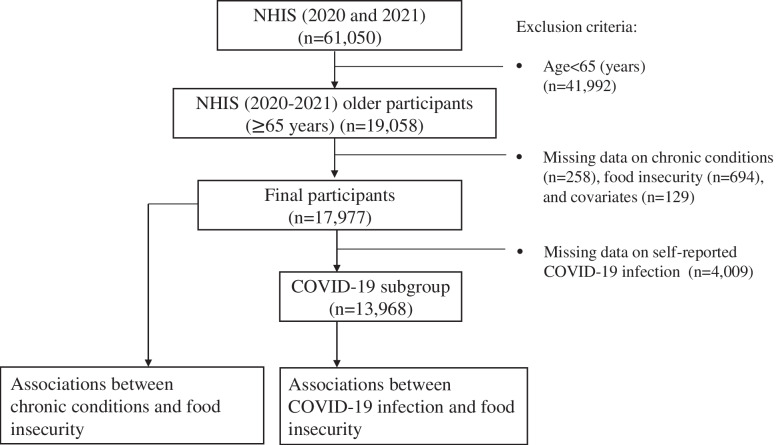
Inclusion and exclusion flow chart, 2020–2021 NHIS Sample *Notes.* NHIS, National Health Interview Survey; COVID-19, coronavirus disease 2019

A total of 61,050 participants (≥ 18 years) completed the survey. In this study, we restricted our sample to participants who were 65 years or older (*n* = 19,058) and excluded those with missing data on chronic conditions (*n* = 258), food insecurity (*n* = 694), and covariates adjusted in the statistical regression models (*n* = 129). This resulted in a final unweighted sample of 17,977 older adults (≥ 65 years). Considering that 22.3% of the older adults (*n* = 4,009) had unavailable data on self-reported COVID-19 infection, we examined the association between COVID-19 infection and food insecurity only in the subgroup with available COVID-related data (*n* = 13,968). The inclusion and exclusion flow chart is shown in Fig. 1. Socio-demographic characteristics of participants were compared between those included and excluded from the COVID-19 subgroup to assess possible selection bias (Appendix Table 1). In the statistical analysis, the final sample was weighted by the two-year sampling weight (two-year weight = annual sampling weights/2) to account for complex survey design and pooled data. Sampling weight for each NHIS cycle was provided by the NHIS analysis program and was calibrated to U.S. Census Bureau population projections and American Community Survey (ACS) one-year estimates for age, sex, race, education, Census division, and Metropolitan Statistical Area status [21]. IRB review was not required since the study relied on a publicly available dataset. Analyses were conducted in 2022. More details about the survey design, dataset, and sampling weights were available on the NHIS website: https://www.cdc.gov/nchs/nhis/2020nhis.htm.

**Table 1:** Socio-demographic characteristics of participants by food security status, 2020–2021 NHIS Sample

Variables	Total^a^ (*n* = 17,977)	High or marginal food security (*n* = 17,329)	Low food security (*n* = 430)	Very low food security (*n* = 218)	*p*-value
**Age (years)**					< 0.0001
65–74	59.5 (10,166)	59.1 (9712)	69.8 (292)	73.3 (162)	
75–84	30.1 (5662)	30.3 (5509)	23.5 (105)	23.0 (48)	
≥ 85	10.4 (2149)	10.6 (2108)	6.7 (33)	3.7 (8)	< 0.0001
**Sex**					< 0.0001
Male	45.1 (7627)	45.4 (7411)	35.4 (137)	43.6 (79)	
Female	54.9 (10,350)	54.6 (9918)	64.6 (293)	56.4 (139)	
**Race/ethnicity**					< 0.0001
Hispanic	8.7 (1170)	8.2 (1062)	22.8 (84)	12.6 (24)	
NH-White	76.0 (14,383)	77.4 (14,047)	38.8 (210)	52.9 (126)	
NH-Black	9.1 (1535)	8.4 (1387)	25.0 (92)	28.6 (56)	
NH-Asian	4.6 (590)	4.5 (555)	10.4 (28)	4.2 (7)	
Others	1.6 (299)	1.5 (278)	3.0 (16)	1.7 (5)	
**Marital status**					< 0.0001
Married	57.9 (8218)	58.7 (8051)	41.4 (126)	28.1 (41)	
Unmarried	7.7 (1602)	7.5 (1519)	13.9 (57)	16.8 (26)	
Widowed	21.3 (4953)	21.3 (4778)	20.6 (107)	26.3 (68)	
Divorced/separated	13.1 (3204)	12.5 (2981)	24.1 (140)	28.8 (83)	
**Region**					< 0.0001
Northeast	18.3 (3140)	18.3 (3040)	21.5 (77)	10.9 (23)	
Midwest	21.0 (4018)	21.2 (3916)	12.9 (62)	17.5 (40)	
South	39.0 (6588)	38.7 (6300)	42.8 (183)	51.4 (105)	
West	21.7 (4231)	21.8 (4073)	22.8 (108)	20.2 (50)	
**Education**					< 0.0001
≤ High school	42.8 (6568)	41.8 (6181)	68.1 (270)	62.6 (117)	
Some college	27.0 (5146)	27.2 (4970)	20.8 (108)	23.4 (68)	
≥ College	30.2 (6263)	31.0 (6178)	11.1 (52)	14.0 (33)	
**Employment**					< 0.0001
Yes	18.4 (3185)	18.7 (3116)	13.8 (47)	11.1 (22)	
No	81.6 (14,792)	81.3 (14,213)	86.2 (383)	88.9 (196)	
**Federal poverty level**^**b**^					< 0.0001
0.00–0.49	1.2 (207)	1.1 (176)	4.3 (21)	3.4 (10)	
0.50–0.99	6.3 (1153)	5.5 (979)	24.8 (112)	25.8 (62)	
1.00–1.99	20.3 (3648)	19.2 (3353)	44.6 (198)	46.9 (97)	
2.00–2.99	18.2 (3271)	18.4 (3182)	13.7 (56)	16 (33)	
≥ 3.00	54.1 (9698)	55.9 (9639)	12.7 (43)	8 (16)	
**Health insurance**					< 0.0001
Yes	98.9 (17,802)	99.0 (17,176)	95.3 (412)	97.7 (214)	
No	1.1 (175)	1.0 (153)	4.7 (18)	2.3 (4)	
**SNAP participation**					< 0.0001
Yes	8.3 (1383)	7.1 (1143)	35.5 (157)	39.3 (83)	
No	91.7 (16,594)	92.9 (16,186)	64.5 (273)	60.7 (135)	

*Notes. NHIS* National Health Interview Survey, *NH* Non-Hispanic, *SNAP* Supplemental Nutrition Assistance Program

^a^Values were expressed as % (n). The number of participants (n) was calculated based on the unweighted sample, and all percentages (%) were based on the weighted sample. *p*-value comparisons across food security categories were calculated using Pearson’s chi-square tests

^b^Federal poverty level was determined by the ratio of family income to the poverty threshold

### Measures of chronic conditions and COVID-19 infection

Chronic conditions measured in NHIS were selected mostly from the list of 20 chronic conditions identified by the U.S. Department of Health and Human Services to foster a standard classification scheme for chronic conditions [22]. In this study, we included physical and mental conditions. Physical health conditions considered: 1) conditions in musculoskeletal and connective tissue including arthritis (arthritis, rheumatoid arthritis, gout, lupus, and fibromyalgia); 2) conditions in the circulatory system including CHD, hypertension, and stroke; 3) endocrine, nutritional, and metabolic diseases including prediabetes and diabetes; 4) conditions in the respiratory system including asthma and chronic obstructive pulmonary disease (COPD); 5) functioning and disabilities [23]. Mental health conditions included clinically diagnosed anxiety or depression disorder. Participants were asked if they had ever been told by a doctor or other health professionals that they had any above chronic condition (yes, no). Functioning and disability were measured using the Washington Group Short Set on Functioning (WG-SS) questions [24]. Details of the WG-SS survey questions are provided in Appendix Table 2. Self-reported COVID-19 infection status based on diagnosis from doctors or health professionals was measured by asking whether a doctor or other health professional ever told the participants that they had or likely had coronavirus or COVID-19 (yes, no). Those who answered “yes” were considered as COVID-19 infected population [21].

**Table 2:** Prevalence of food insecurity in older adults (≥ 65 years) by chronic conditions and COVID-19 infection status, 2020–2021 NHIS sample

	**Total**^**a**^** (*n* = 17,977)**	**High or marginal food security (*n* = 17,329)**	**Low food security(*n* = 430)**	**Very low food security (*n* = 218)**	***p*-value**
**Any chronic conditions**					< 0.0001
No	13.9 (2421)	98.0 (2378)	1.4 (32)	0.5 (11)	
Yes	86.1 (15,556)	95.7 (14,951)	2.9 (398)	1.4 (207)	
**Physical health conditions**					
**Arthritis**					< 0.0001
No	53.0 (9313)	96.7 (9058)	2.5 (175)	0.9 (80)	
Yes	47.0 (8664)	95.2 (8271)	3.0 (255)	1.8 (138)	
**CHD**					< 0.0001
No	85.6 (15,408)	96.3 (14,898)	2.6 (352)	1.1 (158)	
Yes	14.4 (2569)	94.2 (2431)	3.4 (78)	2.4 (60)	
**Hypertension**					< 0.0001
No	39.1 (6990)	97.1 (6808)	2.0 (126)	0.8 (56)	
Yes	60.9 (10,987)	95.3 (10,521)	3.2 (304)	1.6 (162)	
**Stroke**					< 0.0001
No	92.8 (16,691)	96.2 (16,134)	2.6 (375)	1.2 (182)	
Yes	7.2 (1286)	92.8 (1195)	4.2 (55)	3.0 (36)	
**Prediabetes**					< 0.0001
No	75.4 (13,706)	96.5 (13,285)	2.4 (284)	1.1 (137)	
Yes	24.6 (4271)	94.3 (4044)	3.8 (146)	1.9 (81)	
**Diabetes**					< 0.0001
No	80.2 (14,647)	96.7 (14,212)	2.3 (293)	1.0 (142)	
Yes	19.8 (3330)	93.0 (3117)	4.6 (137)	2.4 (76)	
**Asthma**					< 0.0001
No	88.7 (15,867)	96.2 (15,353)	2.6 (346)	1.2 (168)	
Yes	11.3 (2110)	94.2 (1976)	3.7 (84)	2.1 (50)	
**COPD**					< 0.0001
No	89.4 (16,061)	96.3 (15,550)	2.6 (346)	1.1 (165)	
Yes	10.6 (1916)	93.1 (1779)	4.1 (84)	2.8 (53)	
**Disability**					< 0.0001
No	82.1 (14,811)	97.0 (14,426)	2.2 (271)	0.8 (114)	
Yes	17.9 (3166)	91.4 (2903)	5.0 (159)	3.6 (104)	
**Mental health conditions**					
**Anxiety disorder**					< 0.0001
No	88.5 (15,817)	96.6 (15,351)	2.4 (316)	1.1 (150)	
Yes	11.5 (2160)	91.5 (1978)	5.5 (114)	2.9 (68)	
**Depression disorder**					< 0.0001
No	85.3 (15,160)	96.7 (14,735)	2.3 (291)	1.0 (134)	
Yes	14.7 (2817)	91.7 (2594)	5.2 (139)	3.1 (84)	
**COVID-19 infection**^**b**^					< 0.0001
Negative	93.9 (13,252)	95.2 (12,816)	2.6 (297)	1.0 (139)	
Positive	6.1 (716)	96.3 (687)	3.0 (19)	1.8 (10)	

*Notes. NHIS* National Health Interview Survey, *COVID-19* coronavirus disease 2019, *CHD* coronary heart disease, *COPD* chronic obstructive pulmonary disease

^a^Values were expressed as % (n). All percentages (%) were based on the weighted sample and the number of participants (n) was calculated based on the unweighted sample

^b^The prevalence of food insecurity by COVID-19 infection status was calculated within participants with available data on COVID-19

### Measures of food insecurity

The 10-item USDA Food Security Survey Module with a 30-day look-back window was used to measure household food insecurity [21, 25]. Participants were asked to answer questions such as “In the past 30 days, did you or others in your family ever cut the size of the meals or skip meals because there was not enough money for food ?” and “In the past 30 days, did you or other adults in your family worry that the food would run out before you got money to buy more ?”. A full list of the 10 items in the module is provided in Appendix Table 3. Based on the number of affirmative answers, food security was categorized into three levels: high or marginal food security (0–2 items affirmed), low food security (3–5 items affirmed), and very low food security (6–10 items affirmed) [25].

**Table 3:** The Association between chronic conditions or COVID-19 infection and food insecurity in the older adults (≥ 65 years), 2020–2021 NHIS sample

	**Low food security vs. high or marginal food security**	**Very low food security vs. high or marginal food security**
	**AOR (95% CI)**^**a**^	**AOR (95% CI)**
**Physical health conditions**		
Arthritis	1.19 (1.18, 1.20)	1.81 (1.79, 1.82)
CHD	1.59 (1.58, 1.59)	2.40 (2.38, 2.41)
Hypertension	1.28 (1.27, 1.28)	1.41 (1.40, 1.42)
Stroke	1.37 (1.36, 1.38)	2.05 (2.03, 2.06)
Prediabetes	1.35 (1.34, 1.35)	1.51 (1.51, 1.52)
Diabetes	1.49 (1.49, 1.50)	1.74 (1.73, 1.76)
Asthma	1.17 (1.16, 1.18)	1.45 (1.43, 1.46)
COPD	1.42 (1.41, 1.42)	1.76 (1.75, 1.77)
Disability	1.81 (1.80, 1.82)	3.58 (3.56, 3.60)
**Mental health conditions**		
Anxiety disorder	2.28 (2.27, 2.29)	2.43 (2.42, 2.45)
Depression disorder	2.19 (2.18, 2.21)	2.83 (2.82, 2.85)
**COVID-19 infection**^**b**^	1.03 (1.01, 1.04)	1.82 (1.80, 1.84)

*Notes. NHIS* National Health Interview Survey, *COVID-19* coronavirus disease 2019, *CHD* coronary heart disease, *COPD* chronic obstructive pulmonary disease, *AOR* adjusted odds ratio, *CI* confidence interval

^a^AORs with 95% CIs were calculated based on the weighted sample. Age, sex, race/ethnicity, marital status, region, education, employment, federal poverty level, health insurance, and SNAP participation were adjusted in the weighted logistic regression models. Participants without chronic conditions or with negative COVID-19 infection were set as the reference group (AORs = 1). All the significant associations were significant at *p* < 0.0001

^b^The association between COVID-19 infection and food insecurity was also adjusted for the presence of any chronic conditions, in addition to socio-demographic variables above

### Covariates

Socio-demographic factors which may confound the association between health conditions and food insecurity were selected as covariates in the statistical models. Demographic variables included age (65–74 years, 75–84 years, and ≥ 85 years), sex (male, female), race/ethnicity (Hispanic, non-Hispanic White, non-Hispanic Black, non-Hispanic Asian, others), region (Northeast, Midwest, South, West), and marital status (married, unmarried, widowed, divorced/separated). Socioeconomic factors considered education (≤ high school, some college, ≥ college), poverty level indicated by the ratio of family income to poverty threshold (0.00–0.49, 0.50–0.99, 1.00–1.99, 2.00–2.99, ≥ 3.00), health insurance (yes, no), employment (yes, no), and participation in the food-related program (i.e., Supplemental Nutrition Assistance Program (SNAP)). SNAP participants were those who received food stamps in the past 12 months [21].

### Statistical analysis

Baseline characteristics of participants by food insecurity were examined using Pearson’s chi-square tests for categorical variables and ANOVA tests for continuous variables. The prevalence of food insecurity was compared between participants with and without chronic conditions, and between participants with positive and negative COVID-19 infection using Pearson’s chi-square tests. Weighted multinomial logistic regression models were used to determine the association between the individual chronic conditions and food insecurity adjusting for socio-demographic factors. Considering that presence of chronic conditions may affect COVID-19 infection status [26], we also adjusted for the presence of any chronic condition in the association between COVID-19 infection and food insecurity, in addition to socio-demographic factors. Adjusted OR (AORs) with 95% confidence intervals (95% CIs) were reported. Participants without chronic conditions or with negative self-reported COVID-19 infection were set as the reference group (AOR = 1).

In addition to the individual chronic conditions, we examined the association between the number of chronic conditions and food insecurity using weighted multinomial logistic regression models controlling for socio-demographic factors. Besides, the Jonckheere-Terpstra trend test, a non-parametric trend test, was employed to assess whether the severity of food insecurity increased as the number of chronic conditions increased (ordinally categorized as 0–1 condition, 2 conditions, and ≥ 3 conditions). Participants with 0–1 chronic condition were set as reference groups (AOR = 1). Additionally, we reported the estimates from the unweighted sample as sensitivity analyses to test the robustness of the results in the supplementary materials (Appendix Table 5 and Table 6). All analyses were conducted using SAS 9.4 software (version 9.4; SAS Institute Inc., Cary, North Carolina). The statistical significance was defined as two-tailed *p* < 0.05.

## Results

In the current study, 17,977 older adults (≥ 65 years) were included. In the weighted sample (45.1% males and 54.9% females), 2.7% of the older adults lived in low food-secure households and 1.3% lived in very low food-secure households. Table 1 shows the socio-demographic characteristics of participants by food security status. Results of univariate analyses showed that age, sex, race/ethnicity, marital status, region, education, employment, federal poverty level, health insurance, and SNAP participation were significantly associated with food insecurity (all *p* < 0.0001). We also reported the adjusted association between each socio-demographic factor and food insecurity controlling for the other socio-demographic factors in Appendix Table 4. Results based on the weighted sample indicated that younger age, female sex, Hispanic/non-Hispanic Black/non-Hispanic Asian/other races, unmarried, widowed, divorced/separated status, living in the Northeast or South region, lower education level, unemployment, poverty, having no health insurance, and SNAP participation were significantly associated with food insecurity (AOR ranged from 1.02 to 8.57, all *p* < 0.0001).

**Table 4:** The association between the number of chronic conditions and food insecurity in the older adults (≥ 65 years), 2020–2021 NHIS sample

	**% (n)**^**a**^	**Low food security vs. high or marginal food security**	**Very low food security vs. high or marginal food security**	***p-value***_**trend**_^**c**^
		**AOR (95% CI)**^**b**^	**AOR (95% CI)**	
**Number of chronic conditions**				
0–1	36.1 (6410)	Ref	Ref	< 0.0001
2	22.2 (3982)	1.09 (1.09, 1.10)	1.27 (1.26, 1.28)	
≥ 3	41.7 (7585)	1.89 (1.88, 1.90)	4.07 (4.04, 4.10)	
**Number of physical health conditions**				
0–1	39.7 (7124)	Ref	Ref	< 0.0001
2	24.0 (4342)	1.38 (1.38, 1.39)	1.62 (1.60, 1.63)	
≥ 3	36.3 (6511)	1.77 (1.76, 1.78)	3.68 (3.66, 3.71)	
**Number of mental health conditions**				
0–1	93.0 (16,630)	Ref	Ref	< 0.0001
2	7.0 (1347)	2.15 (2.14, 2.16)	2.73 (2.72, 2.75)	

*Notes. NHIS* National Health Interview Survey, *AOR* adjusted odds ratio, *CI* confidence interval, *Ref* reference group

^a^Values were expressed as % (n). All percentages (%) were based on the weighted sample and the number of participants (n) for each group was calculated based on the unweighted sample

^b^AORs with 95% CIs were calculated based on the weighted sample. Age, sex, race/ethnicity, marital status, region, education, employment, federal poverty level, health insurance, and SNAP participation were adjusted in the weighted logistic regression models. Participants with 0–1 chronic condition, 0–1 physical condition, or 0–1 mental condition were set as the reference group (AORs = 1). All significant associations were significant at *p* < 0.0001

^c^Jonckheere-Terpstra test, a non-parametric trend test, was used to assess whether the severity of food insecurity increased as the number of chronic conditions increased

Table 2 presents the prevalence of food insecurity among participants with or without chronic conditions and COVID-19 infection. The results showed that 86.1% of the older participants had at least one chronic condition. The most prevalent chronic conditions identified in older adults were hypertension (60.9%), arthritis (47.0%), prediabetes (24.6%), and diabetes (19.8%). Compared with participants without any chronic condition, the prevalence of low or very low food security was significantly higher in those with at least one chronic condition (low food security: 1.4% in participants without chronic conditions vs. 2.9% in participants with chronic conditions; very low food security: 0.5% in participants without chronic conditions vs. 1.4% in participants with chronic conditions; *p* < 0.0001). Similar trends were observed for all physical and mental health conditions (all *p* < 0.0001).

A total of 13,968 participants had available data on self-reported COVID-19 infection. Socio-demographic characteristics of participants excluded from the COVID-19 subgroup (*n* = 4,009) were not significantly different from those included (all *p* > 0.05) (Appendix Table 1). In the COVID-19 subgroup, 6.1% of the older participants reported a positive diagnosis of COVID-19 infection. Prevalence of low or very low food security was significantly higher in the COVID-19 infected group than the non-infected group (low food security: 2.6% in the negative group vs. 3.0% in the positive group; very low food security: 1.0% in the negative group vs. 1.8% in the positive group; *p* < 0.0001) (Table 2).

The association between chronic conditions, COVID-19 infection, and food insecurity is shown in Table 3. After adjusting for socio-demographic factors, all physical and mental health conditions were significantly associated with low or very low food security (AOR ranged from 1.17 to 3.58, all *p* < 0.0001). The magnitude of the association increased with the increased severity of food insecurity. For example, compared with participants without diabetes, the odds of being low food secure was 49% higher for those with diabetes (95% CI: 1.49, 1.50), while the odds of being very low food secure was 74% higher for diabetic participants (95% CI: 1.73, 1.76). Similarly, significant associations between chronic conditions and food insecurity were also observed with the unweighted sample (Appendix Table 5). Besides, the COVID-infected participants had 3% higher odds of being low food secure (95% CI: 1.01, 1.04) and 82% higher odds of being very low food secure (95% CI: 1.80, 1.84) compared with the non-infected participants controlling for socio-demographic factors. However, the results from the unweighted sample did not suggest any significant association between COVID-19 infection and food insecurity (Appendix Table 5).

**Fig. 2:**
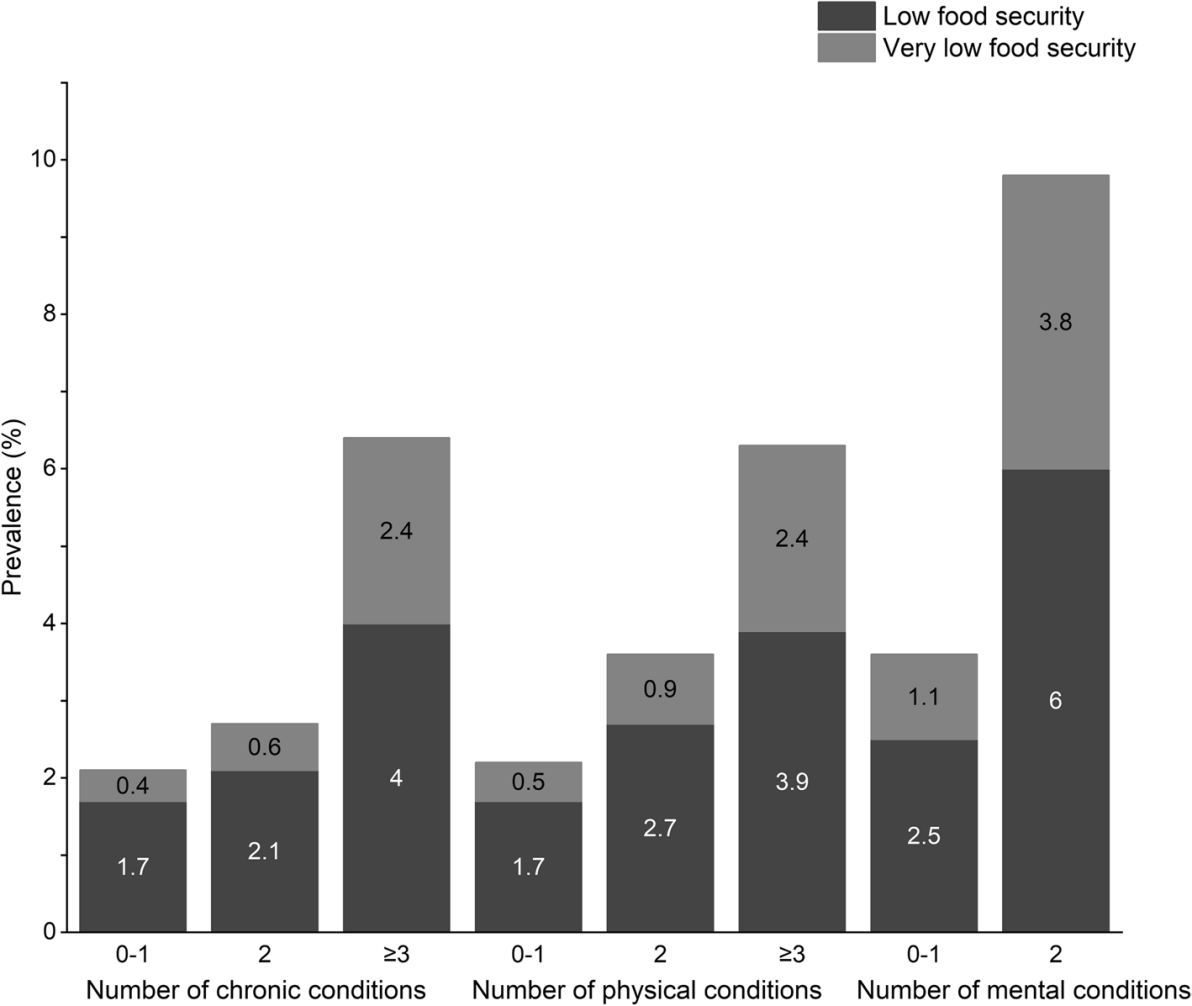
Prevalence of food insecurity by the number of chronic conditions in the older adults (≥ 65 years), 2020–2021 NHIS sample. *Notes.* NHIS, National Health Interview Survey. The prevalence of food insecurity was calculated based on the weighted sample

In addition to the individual condition, we further examined the association between the number of chronic conditions and food insecurity and tested if there was a trend that the severity of food insecurity would increase as the number of chronic conditions increased. Figure 2 displays the prevalence of food insecurity by the number of chronic conditions. As the number of chronic conditions increased, the prevalence of low or very low food security also increased. Similar trends were observed for physical and mental conditions. Table 4 shows the association between the number of chronic conditions and food insecurity. Compared with participants with 0–1 chronic condition, the odds of being low food secure was 1.09 (95% CI: 1.09, 1.10) or 1.89 (95% CI: 1.88, 1.90) times higher for participants with 2 or ≥ 3 chronic conditions, respectively. The odds of being very low food secure was also significantly higher for participants with 2 or ≥ 3 chronic conditions than for those with 0–1 chronic condition (2 conditions: AOR = 1.27, 95% CI: 1.26, 1.28; ≥ 3 conditions: AOR = 3.68, 95% CI: 3.66, 3.71). Results from the trend tests indicated that the severity of food insecurity increased as the number of chronic conditions increased (*p *_*trend*_ < 0.0001). Similar results were observed for physical and mental conditions. Estimates from the unweighted sample were also reported in Appendix Table 6. A significant association between the number of chronic conditions and food insecurity was observed in the unweighted sample comparing those with 0–1 chronic condition to those with ≥ 3 chronic conditions. However, we did not observe significant association when comparing those with 0–1 chronic condition to those with 2 chronic conditions.

## Discussion

Overall, 4.0% of the older participants of the 2020–2021 NHIS lived in food-insecure households. After controlling for socio-demographic factors, the odds of being food insecure was significantly higher for participants with chronic conditions or positive COVID-19 infection than for those without. A dose–response relationship was evident between the number of chronic conditions and the severity of food insecurity.

Data from the 2020 Current Population Survey (CPS) showed that 6.8% of the older adults (≥ 60 years) lived in food-insecure households and 2.6% lived in very low food-secure households, which was slightly higher than the estimates in our study [3]. Different survey time frames (January 2020 to December 2021 in our study vs. December 2020 in CPS), age cutoffs (≥ 60 years in our study vs. ≥ 65 years in CPS), and measures of food insecurity (10-item module in our study vs. 18-item module in CPS) may account for the different prevalence of food insecurity between NHIS and CPS participants. Despite the relatively lower prevalence of food insecurity in older adults (when compared to the general population) and decreasing trend since 2015 [3], the burden of food insecurity was still heavy in this community considering the large absolute number which affected millions of older adults.

The presence of chronic conditions was significantly associated with food insecurity. Our results were consistent with a cross-sectional study with 77,053 Canadian adults which reported that participants with chronic conditions (e.g., asthma, diabetes, back problems, migraines, stomach or intestinal ulcers) had significantly higher odds of being food insecure than those without chronic conditions (AOR ranged from 1.27 to 1.53, *p* < 0.05) [27]. Though the dose–response relationship between the number of chronic conditions and severity of food insecurity was rarely examined, a cross-sectional study with 3,552 low-income US adults identified similar associations between multiple chronic conditions (2–4 conditions and ≥ 5 conditions) and food insecurity (AOR ranged from 2.12 to 3.64, *p* < 0.05) [28]. Besides, our results indicated that poorer mental health was related to food insecurity, which was in line with evidence from other populations such as Puerto Rican adults and Medicare enrollees [29, 30]. A longitudinal study with a 5-year follow-up identified baseline depressive symptoms and perceived stress could significantly increase risks of food insecurity in 517 Puerto Rican adults (45–75 years) [29].

Additionally, we observed a significant association between COVID-19 infection and food insecurity. A similar association was observed in Health and Retirement Study (HRS) in June 2020 with a random sample of 3,212 older participants (≥ 50 years) (AOR = 1.73, 95% CI: 1.03, 2.90) [31]. Considering that most of our participants (81.6%) were not employed and may have already retired, the explanation that the presence of chronic conditions could increase the vulnerability of food insecurity by limiting individuals’ workability and affecting household income may not be established. One possible explanation is that ill health may add additional economic burden and bring an “eat or treat” tradeoff between food and healthcare costs, especially for households with limited budgets. 66% of the respondents in the Hunger in America Study reported choosing between paying for food and medicine or medical care [32]. 1 in 3 chronically ill adults was unable to afford food, medications, or both [33]. Based on the Medication Access report, 43% of the participants cut off expenses on basic needs including food to obtain vital medications in 2020 [34]. Although costs of medication or medical healthcare could be fully or partly covered by health insurance, 8.6% of adults of all ages and 0.7% of older adults (≥ 65 years) were still uninsured in the U.S. in 2020 [35]. Among adults covered by health insurance, those with one or more chronic conditions had 18% to 173% higher odds of having higher amounts of out-of-pocket costs or medical debt than those without any chronic conditions (AOR ranged from 1.18 to 2.73, all *p* < 0.05) [36]. Those additional health costs may force adults with poorer health conditions to choose between food and needed medication or medical treatment, thus rendering their households more vulnerable to food insecurity than those not suffering from ill health. Besides, individuals with poorer health conditions may also be more likely to seek healthier foods which may be expensive compared with healthy individuals, thus making those with ill health more vulnerable to food insecurity.

Though we have argued that chronic conditions or positive COVID-19 infection were associated with food insecurity, reverse causation could not be excluded due to the cross-sectional nation of this study. Though food insecurity was measured within a 30-day look-back window at the time of the survey, specific dates of diagnosis of COVID-19 infection or chronic conditions were not collected in NHIS. Therefore, the temporal relationship between health conditions and food insecurity could not be determined. The association between adverse health conditions and food insecurity is likely to be bi-directional. Adverse chronic conditions of older adults may exert an additional financial burden for households with limited budgets, thus increasing the likelihood of food insecurity. Food insecurity could alternatively increase the risks of adverse health conditions such as diabetes [37], hypertension [38], and mental problems such as stress, anxiety, and depression [39, 40]. Besides, food-insecure individuals may also be more vulnerable to COVID-19 infection due to poorer diet quality [41], insufficient nutrients [42], less practicing social distancing in public, or less engagement in COVID-19 protective behaviors (such as stocking up on essentials, filling prescriptions, and avoiding crowds or larger gathering) [43]. More longitudinal data are needed to fully understand how adverse health conditions may affect food insecurity and whether there is a bi-directional relationship between food insecurity and health status.

Our study was strengthened by using a nationally representative sample of older adults from the NHIS. There were a few limitations of this study. First, causality and the long-term effects of adverse health conditions on food insecurity could not be inferred using the cross-sectional study. Second, measures of chronic conditions and COVID-19 infection were self-reported, therefore differential measurement bias may exist because the status of health conditions may affect accurate reports of food insecurity. Third, lower response rates in 2020 and 2021 NHIS compared to those before the COVID-19 pandemic (2019 NHIS) may underestimate the prevalence of food insecurity in the older population. Fourth, chronic conditions measured in NHIS were limited and did not include chronic conditions such as chronic kidney diseases and cancer. Detailed information on COVID-19 infection (such as variants, specific symptoms, and severity) was not provided in NHIS and could not be controlled for when examining the association between health conditions and food insecurity. Specific employment status (i.e., main job types) was also not adjusted for due to missing data (38.1% (*n* = 12,022) and 38.6% (*n* = 11,391) missing values for main job types in 2020 and 2021 NHIS) [21]. Though participants excluded from the COVID-19 subgroup were not significantly different from those included and the sampling weight has been applied to account for non-response bias, the reduced sample size of the COVID-19 subgroup may still attenuate the statistical power when examining the association between COVID-19 infection and food insecurity in this group. Last, positive COVID-19 infection may pose health challenges for older adults. Failure to adjust for COVID-19 infection due to unavailable COVID-19 infection data (i.e., 22.3% (*n* = 4,009) of the participants in the full sample did not have data on COVID-19 infection status) may overestimate the association between chronic conditions and food insecurity. Unmeasured factors associated with health conditions and food insecurity could still exist and confound the association.

### Public health implications

The presence of chronic conditions or positive COVID-19 infection is associated with food insecurity independent of socio-demographic factors. Health professionals may help identify those at risk and assist them access essential food and financial resources. Allocations of food benefits are necessary for older adults with undesired health conditions. Chronic disease control and management may also help reduce the prevalence and severity of food insecurity in the older community.

## Supplementary Information

**Additional file 1:** **Appendix Table 1.** Comparison of characteristics between participants included and participants excluded from the COVID-19 subgroup, 2020-2021 NHIS sample. **Appendix Table 2.** Measures of Disability in Washington Group Short Set on Functioning (WG-SS). **Appendix Table 3.** Measures of Food Insecurity in NHIS: U.S. Adult Food Security Survey Module (10-Item). **Appendix Table 4.** Socio-demographic characteristics associated with food insecurity in the older adults (≥65 years), 2020-2021 NHIS Sample. **Appendix Table 5.** The Association between chronic conditions or COVID-19 infection and food insecurity in the older adults (≥65 years), 2020-2021 NHIS unweighted sample. **Appendix Table 6.** The association between the number of chronic conditions and food insecurity in the older adults (≥65 years), 2020-2021 NHIS unweighted sample.

## Data Availability

Data described in the manuscript were publicly available at CDC website: https://www.cdc.gov/nchs/nhis/data-questionnaires-documentation.htm.
