# Supplementary material for: The association between chronic conditions, COVID-19 infection, and food insecurity among the older US adults: findings from the 2020–2021 National Health Interview Survey
Source: BMC Public Health. 2023 Jan 27;23:179. doi: 10.1186/s12889-023-15061-8 (PMC9880360; doi:10.1186/s12889-023-15061-8)
Supplement: Supplementary file 1 — Additional file 1: Appendix Table 1. Comparison of characteristics between participants included and participants excluded from the COVID-19 subgroup, 2020-2021 NHIS sample. Appendix Table 2. Measures of Disability in Washington Group Short Set on Functioning (WG-SS). Appendix Table 3. Measures of Food Insecurity in NHIS: U.S. Adult Food Security Survey Module (10-Item). Appendix Table 4. Socio-demographic characteristics associated with food insecurity in the older adults (≥65 years), 2020-2021 NHIS Sample. Appendix Table 5. The Association between chronic conditions or COVID-19 infection and food insecurity in the older adults (≥65 years), 2020-2021 NHIS unweighted sample. Appendix Table 6. The association between the number of chronic conditions and food insecurity in the older adults (≥65 years), 2020-2021 NHIS unweighted sample. [file 12889_2023_15061_MOESM1_ESM.docx]

**Appendix**

**The association between chronic conditions, COVID-19 infection, and food insecurity among the older US adults: findings from the 2020-2021 National Health Interview Survey**

**Appendix Table 1. Comparison of characteristics between participants included and participants excluded from the COVID-19 subgroup, 2020-2021 NHIS sample**

|  | **Included participants**  **(n=13,968)** | **Excluded participants**  **(n=4,009)** | ***p*-value** |
| --- | --- | --- | --- |
| **Age (years)** |  |  |  |
| 65-74 | 56.6 (7911) | 56.3 (2255) | 0.84 |
| 75-84 | 31.5 (4397) | 31.6 (1265) |  |
| ≥85 | 11.9 (1660) | 12.2 (489) |  |
| **Sex** |  |  | 0.14 |
| Male | 42.7 (5967) | 41.41 (1660) |  |
| Female | 57.3 (8001) | 58.59 (2349) |  |
| **Race/ethnicity** |  |  | 0.30 |
| Hispanic | 6.6 (916) | 6.3 (254) |  |
| NH-White | 80.0 (11167) | 80.2 (3216) |  |
| NH-Black | 8.7 (1211) | 8.1 (324) |  |
| NH-Asian | 3.2 (440) | 3.7 (150) |  |
| Others | 1.7 (234) | 1.7 (65) |  |
| **Marital status** |  |  |  |
| Married | 45.8 (6400) | 45.4 (1818) | 0.41 |
| Unmarried | 9.0 (1262) | 8.5 (340) |  |
| Widowed | 27.3 (3811) | 28.5 (1142) |  |
| Divorced/separated | 17.9 (2495) | 17.7 (709) |  |
| **Region** |  |  | 0.20 |
| Northeast | 17.16 (2397) | 18.5 (743) |  |
| Midwest | 22.33 (3119) | 22.4 (899) |  |
| South | 36.8 (5140) | 36.1 (1448) |  |
| West | 23.71 (3312) | 23.0 (919) |  |
| **Education** |  |  | 0.05 |
| $\leq$High school | 36.1 (5043) | 38.0 (1525) |  |
| Some college | 28.5 (3983) | 29.0 (1163) |  |
| $\geq$College | 35.4 (4942) | 33.0 (1321) |  |
| **Employment** |  |  | 0.19 |
| Yes | 17.5 (2447) | 18.4 (738) |  |
| No | 82.5 (11521) | 81.6 (3271) |  |
| **Federal poverty level** |  |  | 0.92 |
| 0.00-0.49 | 1.1 (157) | 1.3 (50) |  |
| 0.50-0.99 | 6.4 (900) | 6.3 (253) |  |
| 1.00-1.99 | 20.2 (2824) | 20.6 (824) |  |
| 2.00-2.99 | 18.2 (2535) | 18.4 (736) |  |
| ≥3.00 | 54.1 (7552) | 53.5 (2146) |  |
| **Health insurance** |  |  | 0.20 |
| Yes | 99.1 (13839) | 98.9 (3963) |  |
| No | 0.9 (129) | 1.1 (46) |  |
| **SNAP participation** |  |  |  |
| Yes | 7.7 (1078) | 7.6 (305) | 0.81 |
| No | 92.3 (12890) | 92.4 (3704) |  |

*Notes.* NHIS, National Health Interview Survey; NH, Non-Hispanic; SNAP, Supplemental Nutrition Assistance Program.

^a^Values were expressed as % (n). *p*-value comparisons across included and excluded groups were calculated using Pearson’s chi-square tests.

**Appendix Table 2. Measures of Disability in Washington Group Short Set on Functioning (WG-SS)**

| **Domains** | **Questions** | **Responses** |
| --- | --- | --- |
| Mobility | Do you have difficulty walking or climbing stairs? | 1. No difficulty  2. Some difficulty  3. A lot of difficulty  4. Cannot do at all |
| Hearing | Do you have difficulty hearing, even when using a hearing aid? |  |
| Seeing | Do you have difficulty seeing, even when even when wearing glasses? |  |
| Cognition | Do you have difficulty remembering or concentrating? |  |
| Self-care | Do you have difficulty with self-care such as washing all over or dressing? |  |
| Communication | Using your usual (customary) language, do you have difficulty communicating, for example, understanding or being understood? |  |

*Notes:* Six domains of disability were measured based on responses to the Washington Group Short Set on Functioning (WG-SS) questions. Participants chose from “no difficulty”, “some difficulty”, “a lot of difficulty” and “cannot do at all” for each question. Participants who had a lot of difficulty or were unable to do activities in any of the above six domains were considered with disability.

**Appendix Table 3. Measures of Food Insecurity in NHIS: U.S. Adult Food Security Survey Module (10-Item)**

| **Items** | **Responses^a^** |
| --- | --- |
| 1. (I/We) worried whether (my/our) food would run out before (I/we) got money to buy more. | 1. Often true  2. Sometimes true  3. Never true |
| 2. The food that (I/we) bought just didn’t last, and (I/we) didn’t have money to get more. | 1. Often true  2. Sometimes true  3. Never true |
| 3. (I/we) couldn’t afford to eat balanced meals. | 1. Often true  2. Sometimes true  3. Never true |
| 4. In the last 30 days, did (you/you or other adults in your household) ever cut the size of your meals or skip meals because there wasn't enough money for food? | 1. Yes  2. No |
| 5. How often did this happen? (If answered “Yes” above) | 1. Almost every month  2. Some months but not every month  3. Only 1 or 2 months |
| 6. In the last 30 days, did you ever eat less than you felt you should because there wasn't enough money for food? | 1. Yes  2. No |
| 7. In the last 30 days, were you very hungry but didn't eat because there wasn't enough money for food? | 1. Yes  2. No |
| 8. In the last 30 days, did you lose weight because there wasn't enough money for food? | 1. Yes  2. No |
| 9. In the last 30 days, did (you/you or other adults in your household) ever not eat for a whole day because there wasn't enough money for food? | 1. Yes  2. No |
| 10. How often did this happen? (If answered “Yes” above) | 1. Almost every month  2. Some months but not every month  3. Only 1 or 2 months |

*Notes:* NHIS, National Health Interview Survey.

^a^Responses of “yes,” “often,” “sometimes,” “almost every month,” and “some months but not every month” were defined as affirmative responses. Based on the summed number of affirmative answers, food security was categorized into four levels: high food security (0 item affirmed), marginal food security (1-2 items affirmed), low food security (3-5 items affirmed), and very low food security (6-10 items affirmed). Participants with high or marginal food security were considered food secure.

**Appendix Table 4. Socio-demographic characteristics associated with food insecurity in the older adults (≥65 years), 2020-2021 NHIS Sample**

|  | **Weighted sample** | **Unweighted sample** |
| --- | --- | --- |
| **Variables** | **AOR (95% CI)^a^** | **AOR (95% CI)** |
| **Age (years)** |  |  |
| ≥85 | Ref | Ref |
| 65-74 | 2.65 (2.63, 2.67) | 2.82 (1.99, 3.98) |
| 75-84 | 1.52 (1.50, 1.53) | 1.50 (1.04, 2.15) |
| **Sex** |  |  |
| Male | Ref | Ref |
| Female | 1.11 (1.11, 1.12) | 1.16 (0.97, 1.39) |
| **Race/ethnicity** |  |  |
| NH-White | Ref | Ref |
| Hispanic | 1.85 (1.83, 1.85) | 1.80 (1.40, 2.31) |
| NH-Asian | 2.40 (2.39, 2.42) | 1.93 (1.30, 2.87) |
| NH-Black | 2.91 (2.89, 2.92) | 2.17 (1.74, 2.71) |
| Others | 1.60 (1.58, 1.62) | 1.58 (0.97, 2.58) |
| **Marital status** |  |  |
| Married | Ref | Ref |
| Unmarried | 1.57 (1.56, 1.58) | 1.27 (0.95, 1.70) |
| Widowed | 1.06 (1.05, 1.06) | 1.10 (0.86, 1.39) |
| Divorced/separated | 1.60 (1.60, 1.61) | 1.62 (1.29, 2.03) |
| **Region** |  |  |
| West | Ref | Ref |
| Midwest | 0.91 (0.90, 0.91) | 0.80 (0.61, 1.06) |
| Northeast | 1.10 (1.10, 1.11) | 0.90 (0.69, 1.19) |
| South | 1.02 (1.02, 1.03) | 1.00 (0.79, 1.23) |
| **Education** |  |  |
| ≥College | Ref | Ref |
| ≤High school | 1.46 (1.45, 1.47) | 1.58 (1.21, 2.05) |
| Some college | 1.20 (1.20, 1.21) | 1.38 (1.04, 1.82) |
| **Employment** |  |  |
| Yes | Ref | Ref |
| No | 1.04 (1.04, 1.05) | 1.14 (0.87, 1.50) |
| **Federal poverty level** |  |  |
| ≥3.00 | Ref | Ref |
| 0.00-0.49 | 6.33 (6.27, 6.39) | 11.17 (6.74, 18.52) |
| 0.50-0.99 | 8.57 (8.52, 8.62) | 10.87 (7.65, 15.45) |
| 1.00-1.99 | 7.42 (7.38, 7.45) | 9.14 (6.72, 12.42) |
| 2.00-2.99 | 3.17 (3.15, 3.19) | 3.76 (2.68, 5.29) |
| **Health insurance** |  |  |
| Yes | Ref | Ref |
| No | 2.40 (2.38, 2.42) | 2.09 (1.27, 3.44) |
| **SNAP participation** |  |  |
| No | Ref | Ref |
| Yes | 2.38 (2.37, 2.38) | 2.30 (1.88, 2.81) |

*Notes.* NHIS, National Health Interview Survey; NH, Non-Hispanic; SNAP, Supplemental Nutrition Assistance Program; AOR, adjusted odds ratio; CI, confidence interval; Ref, reference group.

^a^AORs and 95% CIs were calculated with both the weighted and unweighted sample. The association between each socio-demographic factor and food insecurity (“low food security” and “very low food security” were combined as “food insecurity”) was estimated using binary logistic regression models adjusting for the remaining socio-demographic factors.

**Appendix Table 5. The Association between chronic conditions or COVID-19 infection and food insecurity in the older adults (≥65 years), 2020-2021 NHIS unweighted sample**

|  | **Low secure vs. food secure** | **Very low secure vs. food secure** |
| --- | --- | --- |
|  | **AOR (95% CI)^a^** | **AOR (95% CI)** |
| **Physical conditions** |  |  |
| Arthritis | 1.42 (1.15, 1.75) | 1.57 (1.18, 2.10) |
| CHD | 1.43 (1.10, 1.87) | 2.26 (1.65, 3.11) |
| Hypertension | 1.26 (1.01, 1.58) | 1.41 (1.03, 1.94) |
| Stroke | 1.52 (1.12, 2.06) | 1.91 (1.31, 2.80) |
| Prediabetes | 1.38 (1.11, 1.70) | 1.60 (1.20, 2.12) |
| Diabetes | 1.49 (1.20, 1.86) | 1.69 (1.26, 2.27) |
| Asthma | 1.47 (1.14, 1.91) | 1.77 (1.27, 2.48) |
| COPD | 1.57 (1.21, 2.05) | 1.79 (1.29, 2.50) |
| Disability | 2.03 (1.64, 2.52) | 3.30 (2.48, 4.40) |
| **Mental conditions** |  |  |
| Anxiety disorder | 2.28 (1.80, 2.90) | 2.77 (2.03, 3.78) |
| Depression disorder | 2.22 (1.77, 2.78) | 2.63 (1.96, 3.54) |
| **COVID-19 infection^b^** | 1.04 (0.64, 1.70) | 1.33 (0.68, 2.60) |

*Notes.* NHIS, National Health Interview Survey; COVID-19, coronavirus disease 2019; CHD, coronary heart disease; COPD, chronic obstructive pulmonary disease; AOR, adjusted odds ratio; CI, confidence interval.

^a^Age, sex, race/ethnicity, marital status, region, education, employment, federal poverty level, health insurance, and SNAP participation were adjusted in the logistic regression models. Participants without chronic conditions or with negative COVID-19 infection were set as the reference group (AORs=1).

^b^The association between COVID-19 infection and food insecurity was also adjusted for presence of any chronic conditions, in addition to socio-demographic variables above.

**Appendix Table 6. The association between the number of chronic conditions and food insecurity in the older adults (≥65 years), 2020-2021 NHIS unweighted sample**

|  | **% (n)^a^** | **Low secure vs. food secure** | **Very low secure vs. food secure** | ***p-value* _trend_^c^** |
| --- | --- | --- | --- | --- |
|  |  | **AOR (95% CI)^b^** | **AOR (95% CI)** |  |
| **Number of chronic conditions** |  |  |  |  |
| 0-1 | 35.6 (6410) | Ref | Ref | <0.0001 |
| 2 | 22.2 (3982) | 1.27 (0.91, 1.79) | 1.00 (0.51, 1.64) |  |
| ≥3 | 41.2 (7585) | 2.23 (1.71, 2.90) | 3.18 (2.14, 4.74) |  |
| **Number of physical conditions** |  |  |  |  |
| 0-1 | 39.6 (7124) | Ref | Ref | <0.0001 |
| 2 | 24.2 (4342) | 1.44 (1.06, 1.95) | 1.26 (0.78, 2.06) |  |
| ≥3 | 36.2 (6511) | 2.05 (1.59, 2.64) | 3.10 (2.12, 4.53) |  |
| **Number of mental conditions** |  |  |  |  |
| 0-1 | 92.5 (16630) | Ref | Ref | <0.0001 |
| 2 | 7.5 (1347) | 2.33 (1.78, 3.05) | 2.83 (2.01, 3.98) |  |

*Notes.* NHIS, National Health Interview Survey; AOR, adjusted odds ratio; CI, confidence interval; Ref, reference group.

^a^Values were expressed as % (n). The number of participants (n) and all percentages (%) were calculated based on unweighted sample.

^b^Age, sex, race/ethnicity, marital status, region, education, employment, federal poverty level, health insurance, and SNAP participation were adjusted in the logistic regression models. Participants with 0-1 chronic condition, 0-1 physical condition, or 0-1 mental condition were set as the reference group (AORs=1).

^c^Jonckheere-Terpstra test, a non-parametric trend test, was used to assess whether the severity of food insecurity increased as the number of chronic conditions increased.
